# The evaluation of nocturia in patients with lower urinary tract symptoms suggestive of benign prostatic hyperplasia and the analysis of the curative effect after medical or placebo therapy for nocturia: a randomized placebo-controlled study

**DOI:** 10.1186/s12894-018-0426-4

**Published:** 2018-12-13

**Authors:** Zhigang Xue, Yunhua Lin, Yongguang Jiang, Nengbao Wei, Jinwen Bi

**Affiliations:** 1 Department of Urology, Beijing Anzhen Hospital, Capital Medical University, Beijing, 100029 China; 2 Department of Urology, Beijing Huairou Hospital, Beijing, 101400 China

**Keywords:** Benign prostatic hyperplasia, Nocturia, Nocturnal polyuria, α-Adrenoceptor antagonists

## Abstract

**Background:**

To study nocturia in patients with lower urinary tract symptoms (LUTS) suggestive of benign prostatic hyperplasia (BPH) after medical or placebo treatment.

**Methods:**

Patients with LUTS suggestive of BPH from several community clinics were included. Patients completed the International Prostate Symptom Score (I-PSS) questionnaire and a 3-day voiding diary. Urinalysis, prostate-specific antigen (PSA) measurement, and prostate ultrasonography were performed. Nocturnal polyuria (NP) was defined as a nocturnal urine fraction exceeding one third of the daily urine output in elderly men. A total of 148 outpatients were randomized to drug treatment (tamsulosin) or placebo treatment. After 8 weeks of treatment, they were re-evaluated using a 3-day voiding diary, PSA measurement, prostate volume (PV), I-PSS, etc.

**Results:**

The average I-PSS score was 20.3, storage symptom score was 11.7, voiding symptom score was 8.6, quality of life (QoL) score was 3.7, PV was 40.4 ± 19.4 ml, and nocturnal urine volume (NUV) was 845.7 ± 339.0 ml. The mean frequency of nocturia was 2.3 ± 1.1 per day, and 94% of the patients had a nocturia frequency of more than two times per day. Of these patients, 76.5% had NP. A significant correlation was found between NUV and the amount of water intake at night and 4 h before sleep (*r* = 0.419,*P* = 0.002; *r* = 0.302,*P* = 0.031). Eighty patients were randomized to drug treatment (tamsulosin) and 68 patients were randomized to placebo treatment. The I-PSS score was 16.8 ± 4.9 to 19.3 ± 5.0 (*p* = 0.002), the storage symptom score was 10.3 ± 3.4 to 10.7 ± 3.4 (*p* = 0.007), and the voiding symptom score was 7.5 ± 2.4 to 8.6 ± 2.3 (*p* = 0.003). The frequency of daytime urination was 7.5 ± 2.6 to 8.1 ± 2.6 (*p* = 0.002), maximum urine volume (ml) was 372.8 ± 103.3 to 302.8 ± 119.3 (*p* = 0.007), and morning urine volume (ml) was 280.5 ± 111.7 to 259.5 ± 100.7 (*p* = 0.003). However, the frequency of nocturia score was 2.8 ± 0.7 to 3.0 ± 0.6 (*p* = 0.306) and the nocturnal urine volume (ml) was 800.7 ± 323.0 to 845.7 ± 303.5 (*p* = 0.056), which did not change significantly. There were significant differences between the NP and non-NP groups in the duration of LUTS, first voided urine volume, daytime urination frequency, and the amount of water intake at night and 4 h before sleep.

**Conclusions:**

Among the symptoms of LUTS, the improvement rates for nocturia were the lowest after medical treatment for BPH. The α-blockers did not improve nocturia, which was a common symptom accompanying LUTS suggestive of BPH. Our results showed that the prevalence of NP was 76.5% and that NP was significantly related to the amount of water intake during the evening and before sleep.

**Trial registration:**

ISRCTN registry, Trial registration number (TRN): ISRCTN85509614, Date of registration: 30/10/2018. This trial was registered retrospectively.

## Background

Nocturia, is perceived as one single symptoms of all lower urinary tract symptoms (LUTS) by most men, which is highly prevalent and the most bothersome component [1]. It has led to significant morbidity and, occasionally, mortality. Scholars have pointed out that, this bothersome symptom is related to cause arousal from sleep, and two or more voids per night are usually considered lead to a decreased health-related quality of life [2]. Nocturia could result from many others disease and conditions (including urological and non-urological diseases) [3]. However, the various causes are not differentiated excluded in men with LUTS/BPH, and none of the individual α_1_-adrenoceptor antagonists without subtype selectivity have obvious benefit. The medical therapy have not shown a significant reduction in frequency of nocturia so far. There are also not equal amounts of studies showing positive or negative results on nocturia [4]. For all of these reasons, nocturia is often multifactorial in etiology, and many medical conditions are associated with nocturia. Therefore, we aim to explore nocturia in patients with lower urinary tract symptoms suggestive of benign prostatic hyperplasia, and we analyzed the curative effect after medical therapy for nocturia.

## Methods

### Patients

From January 2015 to May 2016, 148 patients were admitted to Beijing Huairou Community Health Service Center, with a median age of 69.0 years, a mean ± standard deviation (SD) age of 62.1 ± 13.6 years, LUTS/BPH duration of 5.0 ± 3.1 years, and absence of treatment using α-blockers and/or 5α-reductase inhibitors. The common inclusion criteria for all participants were age ≥ 50 years and diagnosis of BPH. All patients provided their written informed consent and the program was accepted by the hospital ethics committee. The exclusion criteria were prostate cancer, PSA > 10 ng/ml, urinary tract infection, nervous system disease, urolithiasis, medical therapy that could affect the function of urination, prostatic surgery, or pelvic surgery.

### Methods

Based on the literature [5], we estimated that 74 patients will need to be recruited in order to detect a clinically significant difference of 25% between the 2 groups (with a power of 80%). Based on past clinical trial experience, we estimated that 20% of randomized participants will be lost in follow up, therefore 88 patients will need to be recruited, and 170 participants who will complete the item in our study.

The patients included in the study were randomly divided (1:1) into two groups (drug treatment and placebo treatment) by use of a computer generated random number table. But some patients of the two groups had lost in follow-up because of financial status, phone lost, language barriers, individual patient factors, etc.

At last all 148 patients with LUTS suggestive of BPH were divided into two random groups, which either received α-adrenoceptor antagonist (tamsulosin 2 mg qd po) or placebo for 8 weeks. IPSS, QoL index questionnaire, 3-day frequency volume charts (FVCs), PSA, urinalysis, PV, and uroflowmetry (Qmax) were assessed before and after medical therapy. Serum PSA and urinalysis were evaluated at Beijing Huairou Hospital. The specific instrument for the evaluation of nocturia was a 1000 ml graduated glass. This study was approved by the ethics committee of Beijing Huairou Hospital. Each patient was informed of the study and signed an informed consent form and had the right to withdraw from the study at any time.

### Nocturia evaluation index

Nocturia, specifically, was the number of voids recorded during the nighttime [3]. The first morning void was excluded from the count because it is not followed by sleep. Nocturnal urine volume describes the amount of urine excreted during the nighttime and includes the volume of the first morning void because this urine has been produced during the nighttime [3]. Nocturnal polyuria is an abnormally large urine volume produced during the nighttime. The ICS classifies nocturnal polyuria as the nocturnal urine volume divided by the 24-h urine volume (i.e., nocturnal polyuria index, NPi) [3]. The ICS defines nocturnal polyuria as NPi > 33% in the elderly [3].

### Statistical analysis

SPSS 22.0 statistical software was used for data analysis. All variables were expressed as the mean ± SD values. The numerical data were compared using the unpaired *t*-test. An analysis of the difference in the number of patients with nocturia before and after treatment was performed using McNemar’s test. A multivariate analysis was performed. A *P-value* of 0.05 or less was considered significant.

## Results

### The clinical index and results of 3-day **frequency-volume charts** from patients with LUTS/BPH (Table 1)

#### The relationship between NUV, NPi, and evening drinking volume 4 h before bedtime drinking volume and other indices (Fig. 1 and Fig. 2)

The relationship between NUV and evening drinking volume (*r* = 0.419, *p* = 0.002) and 4 h before bedtime drinking volume (*r* = 0.302, *p* = 0.031) were related. NPi and maximum urination volume (*r* = 0.440, *p* = 0.001) and morning urine volume (*r* = 0.445, *p* = 0.001) were related, but there was no correlation between NPi and prostate volume, age, LUTS duration, or I-PSS score.

**Table 1:** Clinical indices and nocturia-related parameters in patients with BPH

Variable	Median (mean ± SD)
Number of patients	148
Age (years)	69.5 ± 5.6
LUTS/BPH duration (years)	6.3 ± 3.1
I-PSS	20.3 ± 5.0
Storage symptom score	11.7 ± 3.4
Voiding symptom score	8.6 ± 2.3
QoL score	3.7 ± 1.0
Nocturia score	3.0 ± 1.0
Serum PSA (ng/ml)	2.0 ± 1.7
Prostate volume (ml)	40.4 ± 19.4
NPi	0.43 ± 0.12
24-h drinking volume (ml)	1565.4 ± 624.8
4-h before bedtime drinking volume (ml)	123.9 ± 109.9
Frequency of nocturia	3.3 ± 1.1
Frequency of daytime urination	8.1 ± 2.6
Maximum urination volume (ml)	352.8 ± 119.3
Morning urine volume (ml)	260.5 ± 109.7
24-h total urine volume (ml)	1996.5 ± 637.5
Nocturnal urine volume (ml)	845.7 ± 339.0

*LUTS* lower urinary tract symptoms, *BPH* benign prostatic hyperplasia, *PSA* prostate-specific antigen, *I-PSS*, international prostate symptom score, *NPi* nocturnal polyuria index, *QoL* quality of life

**Fig. 1:**
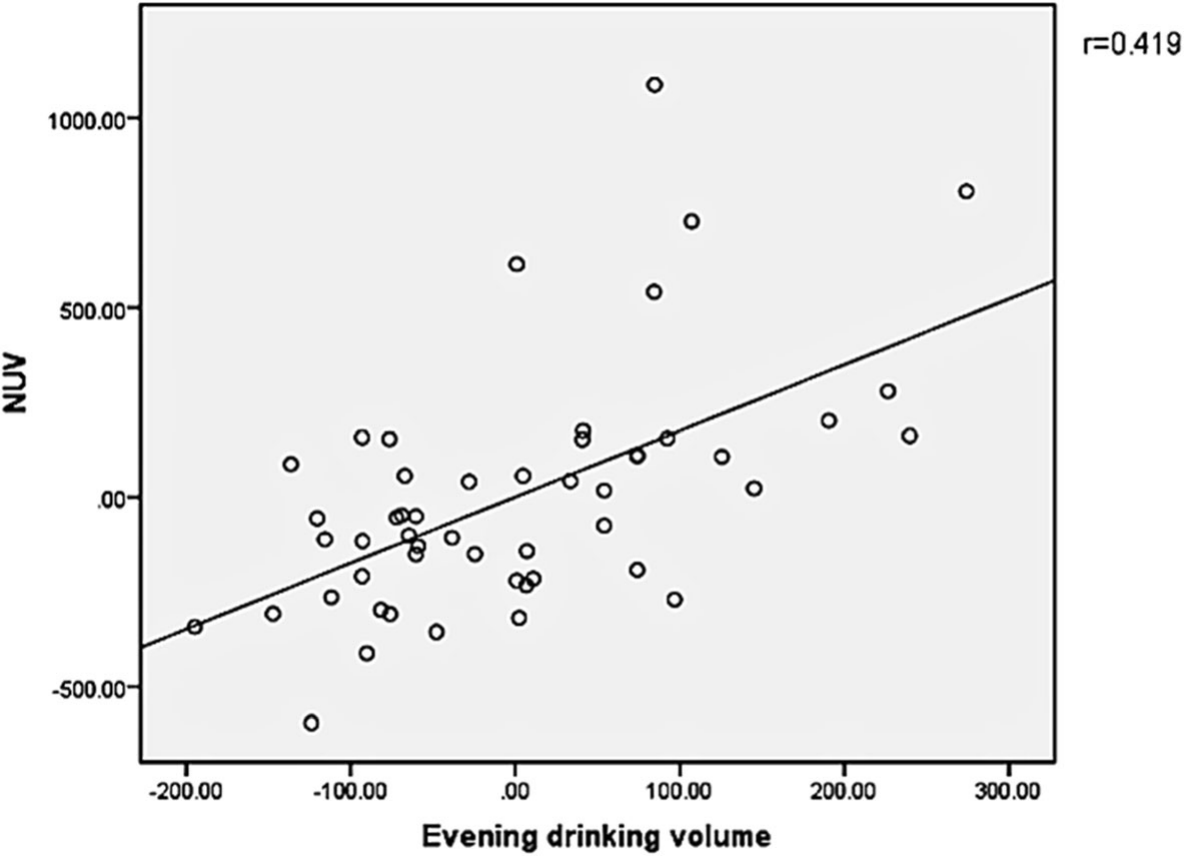
The relationship between NUV and Evening drinking volume. 1) X axis caption: “Evening drinking volume (ml)”. 2) Y axis caption: “NUV (ml)”

**Fig. 2:**
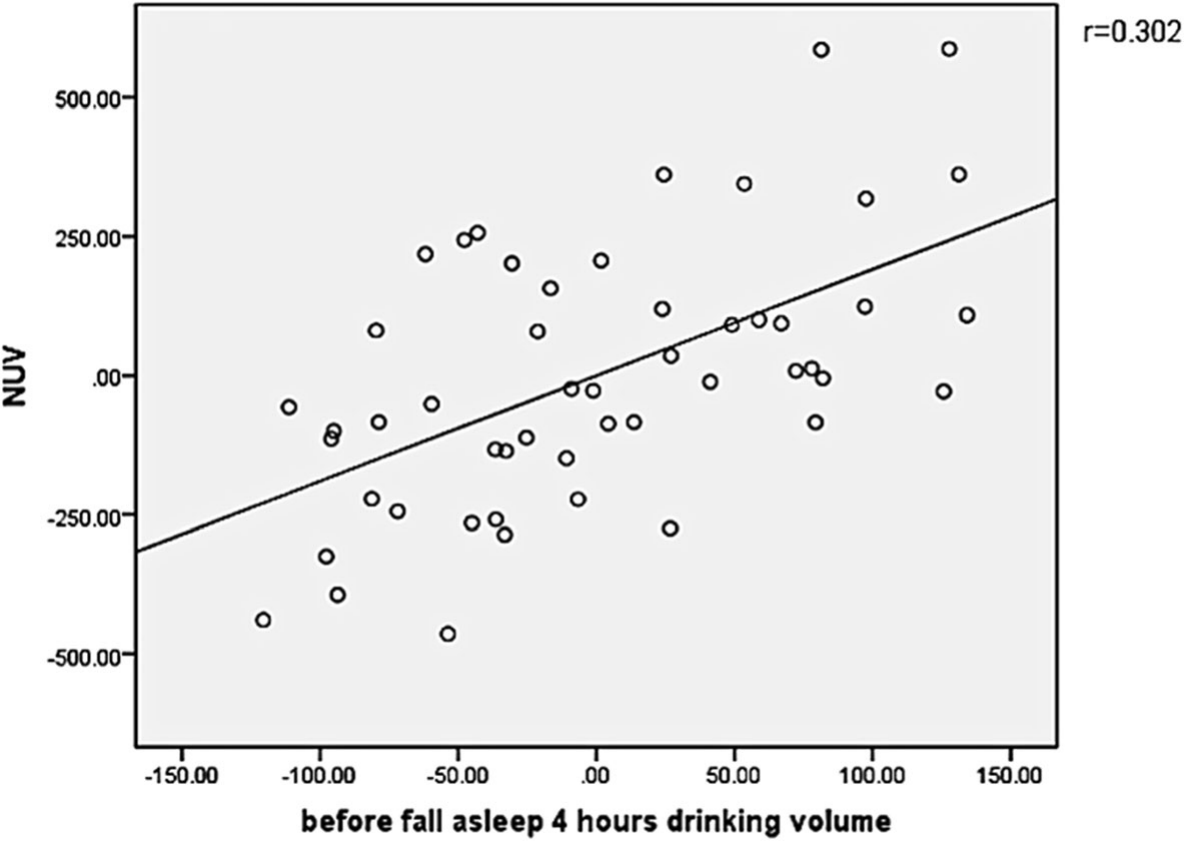
The relationship between NUV and before fall asleep 4 h drinking volume. 1) X axis caption: “4-h before bedtime drinking volume (ml)”. 2) Y axis caption: “NUV (ml)”

#### The comparison of evaluation indices between drug treatment and placebo treatment groups for patients with BPH (Table 2)

Eighty patients were randomized to the drug treatment (tamsulosin) and 68 patients were randomized to the placebo treatment. The I-PSS score, storage symptom score, voiding symptom score, quality of life score, frequency of daytime urination, maximum urine volume, and morning urine volume were statistically significant between both groups. However, the frequency of nocturia score and nocturnal urine volume did not change significantly.

**Table 2:** Comparison of evaluation indices after treatment (drug or placebo) in patients with BPH and nocturia

Variable	Drug reatment	Placebo Treatment	*P value*
Number of patients	80	68	
Age (years)	69.5 ± 5.6	68.5 ± 5.3	0.450
I-PSS	16.8 ± 4.9	19.3 ± 5.0	0.002
Storage symptom score	10.3 ± 3.4	10.7 ± 3.4	0.007
Voiding symptom score	6.5 ± 2.4	8.6 ± 2.3	0.003
QoL score	3.1 ± 0.6	3.8 ± 1.0	0.005
Serum PSA (ng/ml)	2.2 ± 1.7	2.1 ± 1.6	0.355
Prostate volume (ml)	39.4 ± 19.4	41.4 ± 20.1	0.235
Qmax	14.0 ± 2.4	16.5 ± 2.6	0.043
24-h drinking volume (ml)	1465.4 ± 624.8	1505.4 ± 610.8	0.356
4-h before bedtime drinking volume (ml)	115.8 ± 100.9	106.6 ± 97.9	0.452
Frequency of nocturia	2.8 ± 0.7	3.0 ± 0.6	0.306
Frequency of daytime urination	7.5 ± 2.6	8.1 ± 2.6	0.002
Maximum urination volume (ml)	372.8 ± 103.3	302.8 ± 119.3	0.007
Morning urine volume (ml)	280.5 ± 111.7	259.5 ± 100.7	0.003
24-h total urine volume (ml)	1965.5 ± 623.5	1896.5 ± 637.5	0.231
Nocturnal urine volume (ml)	800.7 ± 323.0	845.7 ± 303.5	0.056

*LUTS* lower urinary symptom, *PSA* prostate-specific antigen

#### Data statistics between the NP group and the non-NP group (Table 3)

In the indices of age, 24-h drinking volume, bladder function, PV, and PSA level, there were no significant differences between the groups. While in LUTS duration, nocturnal urine volume, morning urine volume, and daytime voiding frequency, the differences between the two groups were statistically significant.

**Table 3:** Comparison of evaluation indices in NP and non-NP patients

Variable	Median (mean ± SD)		
	NPi ≥0.33	NPi < 0.33	*P value*
Number of patients	113	35	
Percentage (%)	76.50	23.50	
Age (years)	69.6 ± 6.0	69.1 ± 4.0	0.746
LUTS duration (years)	5.8 ± 2.7	7.9 ± 4.0	0.043
24-h drinking volume (ml)	1931.9 ± 680.6	2206.5 ± 429.1	0.195
4-h before bedtime drinking volume (ml)	140.8 ± 135.8	132.9 ± 102.4	0.029
Nocturnal urine volume (ml)	922.4 ± 342.6	596.5 ± 168.2	0.003
Morning urine volume (ml)	278.3 ± 116.5	202.5 ± 55.0	0.004
Frequency of daytime urination	7.4 ± 2.2	10.6 ± 2.4	0.005
Bladder capacity index	0.8 ± 0.6	0.8 ± 0.6	0.963
Maximum urination volume (ml)	369.1 ± 126.0	299.4 ± 75.7	0.076
Prostate volume (ml)	41.9 ± 25.3	39.5 ± 24.8	0.198
Peak flow rate (ml/s)	13.2 ± 10.0	13.9 ± 9.9	0.12
Residual volume (ml)	21.8 ± 20.0	20.6 ± 18.7	0.087
Serum PSA (ng/ml)	4.7 ± 4.1	4.5 ± 4.0	0.102

*NPi* nocturnal polyuria index

## Discussion

Nocturia, one of the most bothersome symptoms of LUTS, has been the focus of a high volume of rapidly evolving research. The purpose of this study was to describe the relevant recent research in the field of nocturia in China, with particular emphasis on its evaluation and management. Nocturia is a complicated clinical entity that is often multifactorial in etiology. Experts of the International Continence Society (ICS) define nocturia as the general complaint when an individual (independent of age, gender, cause(s) and associated bother) must wake up at night one or more times to void [1, 2].

Epidemiological studies showed that the prevalence of nocturia in the < 30-year-old population was approximately 3%, in the 60- to 69-year-old population was 30%, and in the > 70-year-old population was 40% [6]. The survey regarding > 60-year-old men in the United States demonstrated that up to 65.2% of old men would get up at night to urinate, of whom 25% of elderly males woke up at night to urinate ≥2 times [7]. A Chinese questionnaire study showed that in patients living in the national scope, the storage symptoms of I-PSS were the most troubling symptoms of BPH in patients, in which nocturia was the most affecting [8]. Schatzl reported that more than 60% of old people thought nocturia would negatively affect their quality of life [6]. Recently, a study showed that the mortality rate of the elderly with nocturia was significantly higher (more than 3 times) than that of the elderly without nocturia (less than 3 times) [9].

Nocturia has been identified as the leading cause for sleep disturbance and sleep fragmentation; it causes daytime fatigue, impacts daily activities, and deteriorates psychomotor performance, cognitive function, and mood [10–12]. Nocturia can also cause depression and immunosuppression, and it increases the vulnerability for cardiovascular diseases and the development of diabetes mellitus [11, 13–15].

In these analyses, LUTS/BPH patients with nocturia had some urinary system diseases, such as lower urinary tract obstruction and overactive bladder, as well as cardiovascular diseases, diabetes, diabetes insipidus, and other awakening factors of urination (such as anxiety and sleep disorders). In this study, patients had hypertension, coronary heart disease, diabetes, and other diseases. Thus, nocturia may be caused by the combined effects of a variety of conditions. Kupelian et al. demonstrated that while increased odds of the metabolic syndrome were observed with mild to moderate degrees of nocturia [16]. In this way, the etiology of nocturia, especially, nocturnal polyuria can be further analyzed by evaluating LUTS patients with metabolic syndrome.

BPH is a common disease in elderly men, not only with voiding symptoms such as dysuria but also with storage symptoms such as urinary frequency, urgency, and nocturia. Clinical studies showed that the application of α-blockers and 5α-reductase inhibitors could effectively relieve lower urinary tract symptoms caused by BPH, but the efficacy in improving nocturia was not obvious [17, 18].

Tacklind thought that doxazosin and terazosin were significantly more effective than finasteride in improving nocturia, while no difference was found with tamsulosin [19]. This lack of specificity precluded prostatic surgery, such as transurethral resection of the prostate (TURP), from being the first-line therapy for nocturia. The symptoms of nocturia could not be improved in most patients. Nonetheless, various groups had reported decreases in nocturia after surgery in specific patient populations. In another study, Wada et al. evaluated the effect of TURP on nocturia in patients with LUTS/BPH. Nocturnal voids reduced from 3.0 to 1.9 per night after surgical treatment, which showed significant difference [5]. Yoshimura et al. reported that 505 cases of patients with BPH after drug treatment of tamsulosin and TURP surgery decreased the frequency of nocturia in patients by 17.9 and 32.2%, respectively, but the efficacy in improving nocturia was not obvious [20]. Additional studies were warranted to validate and substantiate these findings. Though such treatments were unlikely to benefit most patients with nocturia, they could be tried in select cases.

In our study, 80 patients were randomized to drug treatment (tamsulosin) and 68 patients were randomized to placebo treatment. The scores and the data (include I-PSS score, storage symptom score, voiding symptom score, quality of life score, frequency of daytime urination, maximum urine volume, and morning urine volume) were all statistically significant. However, the frequency of nocturia score and the nocturnal urine volume did not change significantly. The traditional tool for evaluating nocturia was the I-PSS in nocturia subtype score, which was not described in detail [21]. Whatsmore, the important and detailed information of nocturia may not be fully captured by the I-PSS questionnaire alone. Michel et al. thought that the I-PSS (QoL and Nocturia score) can’t show how nocturia decreases sleep quality [22]. Therefore, a thorough evaluation of any treatment of nocturia requires using a method for measuring the treatment impact on sleep quality and QoL [18]. The international consultation on incontinence questionnaire (ICIQ) N-QoL is developed by Abraham et al., and it is a validated questionnaire that can measure the deterioration of health-related quality of life (HRQoL) due to nocturia in general [23]. In our study, we also found it was just a basic assessment of the patient’s own quality of life use I-PSS (QoL) score. On the one hand we worried about patients’ coordination and understanding, on the other hand we ignored the importance of nocturia to quality of life. The (ICIQ) N-QoL et al. should be used to evaluate nocturia on quality of life in further study.

We used a 3-day urinary frequency voiding diary, including a 24-h urination, nocturnal urine volume, urine volume, total sleeping time, and amount of water intake for a detailed assessment of patients with nocturia to understand the correlation between nocturia and other factors. Therefore, the data that was obtained was in detail.

Koseoglu et al. reported 58 cases of patients with BPH, of whom 95% had NP. The frequency of nocturia was 2.73 ± 1.44 times/day, which confirmed that patients with BPH and NP had a relationship between LUTS duration and 3-h drinking of water before falling asleep [24]. In this study, patients with BPH were diagnosed as BPH/LUTS, which led to the decrease of a certain degree of bladder function, and all patients received treatment (drug treatment such as tamsulosin or placebo treatment). To some extent, medications could alleviate lower urinary tract symptoms in patients with BPH, including storage symptoms such as urinary frequency and urgency. However, according to the analysis on 3-day voiding diary results, NP was reported in 76.5% of patients with nocturia, which is lower than that reported for 95% of the patients with NP type nocturia. However, the proportion is still higher than other types of nocturia. Our results showed that there was no association between nocturnal urine volume and LUTS duration, PV, serum PSA, or I-PSS. However, there was a significant correlation between the amount of drinking water at night (*r* = 0.419, *p* = 0.002) and before falling asleep 4-h drinking water (*r* = 0.302, *P* = 0.031). Hence, we thought that the improvement of α-blockers treatment for nocturia was poor.

In this study, between the NP group and the non-NP group, there was a significant difference in LUTS duration, maximum urine volume, morning urine volume, nocturnal urine volume, daytime urination, and 4-h before falling asleep drinking water volume. The frequency of daytime micturition and LUTS duration were the indices between the NP group and the non-NP group. A long disease history and more frequent daytime urination were considered the non-NP type, and α-blocker drug treatment could improve this type of nocturia. Nocturia could also be alleviated by treating the other causes, such as increased water drinking before going to bed.

This study had some limitations, including a small sample size and the assessment with regard to short-term use of medication. Future studies of patients with LUTS/BPH and nocturia should, therefore, also include nocturia-specific instruments to correctly classify the underlying pathologies for nighttime voiding, and hence, provide a rationale for the treatment of all LUTS/BPH causes.

## Conclusions

In conclusion, patients with lower urinary tract symptoms improved, to some extent, after receiving medication. However, the nocturia was not obviously improved and the incidence was still high. The patients could not achieve satisfactory results, and NP was highly prevalent among patients. The correlation between the frequency of nocturia and nighttime water intake and drinking water before going to bed were significant. We could control water intake to ease nocturnal urination.

## Abbreviations

BPH: Benign prostatic hyperplasia
I-PSS: International Prostate Symptom Score
NP: Nocturnal polyuria
Npi: Nocturnal polyuria index
